# Past, present and future: experiences and lessons from telehealth projects

**Published:** 2007-12-04

**Authors:** Laurent Elder, Michael Clarke

## Abstract

Information communications technology has been a focus of the work of the International Development Research Centre (IDRC) since 1970, when this organization was formed in Canada with the goal of helping to improve the health of people in developing countries (http://www.idrc.ca). In this article, we focus on the field of telemedicine in developing countries and its role in improving health, using examples from the experience of the IDRC.

Information communications technology has been a focus of the work of the International Development Research Centre (IDRC) since 1970, when this organization was formed in Canada with the goal of helping to improve the health of people in developing countries (http://www.idrc.ca). In this article, we focus on the field of telemedicine in developing countries and its role in improving health, using examples from the experience of the IDRC.

## Past: the difficult work of pioneers

One of the authors of this article (LE) was involved in a pioneering project on telemedicine in Uganda in 2000. The aim of this project was to enhance access to health services using telemedicine, such that consultations with doctors who worked in larger hospitals in Mulago and Butabika could be obtained for patients who did not live near a hospital. The project focused on cholera, malaria and HIV/AIDS. Further goals were to disseminate health information and build a continuing medical education program. Finally, the project was meant to document lessons on these different activities.

These efforts were quite typical of activities that focused on health and on information and communications technology (ICT) at the time: overly ambitious, lacking in adequate capacity and planning, but spurred by the drive and determination of project proponents, who went on to use their experiences to become champions of telehealth in their countries. What actually happened? As was typical of early telehealth projects in Africa, the project was faced with challenges related to procuring appropriate equipment and setting up infrastructure, as well as difficulties in achieving connectivity. Disappointingly, the project never actually made an online consultation between Kampala and the rural health centres, and it would be remiss to say that it resulted in any direct beneficial health outcomes for the rural population.

Nevertheless, the project did offer some valuable lessons for future e-health projects. It was in many respects ahead of its time, and set the stage for more successful e-health projects in Uganda, such as the Uganda Health Information Network and a subsequent telehealth project in Mengo. Indeed, with the support of Memorial University in St. John’s, NL, the project helped train and mentor numerous staff in telehealth activities; it further helped focus the attention of the government on rural health problems and potential solutions; and it developed educational materials that are in use today.

The project provided significant insights and learning. First, it helped the organization better understand the challenges of supporting telehealth projects in Africa and helped define some of the key questions it would try to answer. Key among these was a better understanding of how appropriate local capacities, both technical and institutional, should be built, Second was the need to focus on the “e-readiness” of the country, particularly with regard to the availability of equipment, cost of access and an enabling regulatory environment. (E-readiness refers to the state of a country’s ICT infrastructure and the ability of consumers, businesses and governments to use ICT to their benefit.) Finally, this experience prompted greater consideration about the key underlying question: Is telehealth a viable means of solving health problems in developing countries?

In this case, cost–benefit analyses had not been done and health outcomes had not been measured, in large part because these efforts had been lost in the challenges to implement the pilot project. All these lessons helped shape future thinking about supporting the development of effective health applications. However, it is also of interest to examine some of the lessons from  programming in Asia on telehealth to demonstrate how lessons coalesced from one region to the other, despite having been implemented through separate programs.

The objectives of the Impact of Remote Telemedicine in Improving Rural Health project in India, part of the Pan Asia Networking (PAN) project, were to study the impact of remote telemedicine in selected villages in India. The activity specifically aims to conduct, with the help of n-Logue, an Internet service company in India, a low-cost medical kit called ReMeDi, which the manufacturer describes as a “Medical Data Acquisition Unit that captures multiple parameters,” i.e., temperature, ECG readings, blood pressure, pulse rate, heart and lung sounds and oxygen saturation (Manufacturer's page).

The telemedicine program can work in conjunction with a rural kiosk and transmit medical information remotely to a doctor in an urban centre. Once the service was launched, there was a spike in the number of visitors to the kiosk. After the initial interest, however, the number of visitors dropped precipitously to a few regular, repeat visitors. The drop was explained by the following factors: “Kiosk Operator’s ability to administer the kit properly, acceptability by the villagers, identification of the Kiosk in a place where medical care is already dispensed, lack of awareness of the service, distance of the doctor from the village, and availability of competing services such as Registered Indian Medical Practitioners Primary Health Centres, local doctors, etc.”^1^ Although the project faced challenges with respect to sustainability, it was, contrary to the Ugandan experience, able to function as a working telemedicine project. However, despite the activity’s stated objective of understanding telemedicine’s “impact,” no findings were documented with regard to health outcomes.

In Indonesia, the Development of ICT-based Telemedicine System for Primary Community Health Care in Indonesia project used existing Internet technology to enhance PC-based medical stations and pilot-tested a telemedicine application. The pilot network consists of six medical stations within community health centres and a station for each referral hospital, health office and test laboratory. The pilot found that human resource capacity-building — in particular, training to facilitate the adoption of computer and telemedicine technology — required significantly more time than expected. The project therefore demonstrated the important role that human resource development plays in the sustainable implementation of ICT-based telemedicine systems. However, as before, no findings were documented on the actual effect the pilots had on people’s health or on health systems.^2^

What parameters were to be used for evaluating programs? Textbox 1 lists those factors that were felt to be relevant and important according to a report, commissioned by IDRC, that unfortunately ranked all projects “low” with respect to demonstrated health benefits. Common deficiencies included a lack of planning and health needs assessment, a need for sustainability planning, difficulty in the management of change, and a need for better evaluation, dissemination of findings, and knowledge transfer to influence policy-making. General concerns included surrounding open-source versus proprietary software and the need to develop capacity with respect to technical knowledge and expertise.^2^

## Present: from adversity come ideas

Understanding the needs of the local environment is crucial to successful, sustainable projects. The IRDC’s Application of ICT in the HIV-AIDS response in Eastern and Southern Africa project (link) illustrated this. This project studied how ICTs had been used in Uganda, Kenya, Tanzania, South Africa and Botswana to address health and development challenges brought about by HIV/AIDS. After having done an extensive literature review, the project proponents undertook a wide-scale electronic survey of individuals and organizations involved in HIV/AIDS issues. Of particular interest was an in-depth impact assessment undertaken in Tanzania and South Africa with 990 respondents.

A comparison of respondents from the two countries showed, as expected, that most received information on antiretroviral treatment (ART) from traditional media. However, increased access to information technology in South Africa resulted in 30% of respondents receiving information from cellphones (versus 10% of respondents in Tanzania). Hence the assumption is that, as access to mobile telephony and the Internet rises in Africa, so will the number of people accessing health information through these technologies.

Moreover, according to the survey, illiteracy was the most important barrier to the use of ICTs in both South Africa and Tanzania. The results echo previous research that showed that illiteracy and localization issues are among the most important factors challenging the more widespread use of ICT solutions.

According to the survey on the effectiveness of ICTs, it was perceived that radio, print and television, as well as face-to-face meetings, were “extremely effective” media. The majority of respondents “didn’t know” whether computers, email and the Internet could be effective. Strangely, almost 9% saw the Internet as “harmful” (the highest percentage in that category). Although one can question the methodology of a perception questionnaire as well as the terms used —What do “harmful” or “extremely effective” actually mean? — one cannot deny that conventional communication methods are still perceived as the most widely used modes of information transmission.

The authors of the AfriAfya (African Network for Health Knowledge Management and Communication) study conclude that the best practices for using ICTs in the fight against HIV/AIDS were (1) use of mobile phones and SMS; (2) ICTs for up-to-date HIV management information; (3) ICTs for mobilization; (4) combination of different ICTs; and (5) telephone counselling. They also pointed out that the use of “modern” ICTs is still very limited, but that there is huge potential; that because institutions and health workers remain reliant on “conventional” ICTs there is a need to integrate both “modern” and “conventional” to get the best results; and, perhaps most important, that changing perceptions and behaviours requires careful planning and patience.

Similarly, Acacia’s 2006–2011 prospectus finds that the impact of ICTs has been constrained by the fact that access to them at the front lines of health care in rural areas has been generally non-existent. However, the rapid expansion of mobile telephony into urban and rural areas in Africa is seen as having brought about new opportunities for access and innovation in the use of ICTs to facilitate the delivery of health care.

Although most mobile infrastructure in Africa is too slow and expensive for connecting computers to the Internet, low bandwidth communication applications have emerged that use mobile phones or personal digital assistants (PDAs) such as Palm Pilots to connect via mobile networks. Indeed, “while information designed and formatted for the World Wide Web is generally too bandwidth intensive to be transmitted over mobile networks, the information itself, properly formatted for small devices, takes up very little bandwidth.” PDAs and smart phones are also seen as more advantageous because of their robustness (no moving parts), their relative affordability, and their ability to be maintained “in areas with little or no electricity infrastructure through the use of solar power rechargers” (link).

Of particular interest, therefore, is the fact that the Acacia program, given that mobile telephony and PDAs are increasingly pervasive in Africa and have the potential to play an important role there, has focused much of its current project support on that theme (link). Examples of mobile-enabled health applications supported by Acacia are listed in Textbox 2.

According to the Pan Asia Networking Prospectus, health is the area where ICTs are likely to have the most direct positive impact in improving the well-being of Asian communities. However, the prospectus also affirms that the first generation of largely donor-driven “telemedicine” projects has generally had only a marginal impact on people’s health. Indeed, many of the technologies previously developed and tested were too expensive to be widely adopted in resource-poor settings. Much like Acacia, PAN sees the advent of more pervasive technologies, such as mobile phones and PDAs, as a new generation of health applications that have actually made a demonstrable difference. As mobile telephony use in Asia is more widespread than in Africa, it is clear that the potential for these types of applications is significant in Asia.

PAN’s strategic document also emphasizes that more research is needed to gauge which applications and projects in the area of health have made a difference, to understand why they have or have not been successful and, when warranted, to scale them up. However, the fast pace of innovation in both ICTs and health research means that there is also a need to develop, implement and evaluate new applications, particularly in the area of demographic surveillance of disease incidence and medical compliance, using new technologies such as mobile phones. According to the prospectus, another area that has recently come to the forefront in Asia is the issue of pandemics. First severe acute respiratory syndrome (SARS) and now the potential for an Avian flu pandemic are perceived as serious threats to the health of Asian populations as well as the rest of the world. A key to mitigating the spread of these infectious diseases is to ensure that data on outbreaks are captured and communicated to the relevant experts in real time. ICTs can play a critical role in helping to prevent or control pandemics, although more research and experimentation need to be done to identify the most appropriate and cost-effective means of developing health communications processes in rural and remote areas, where many of these outbreaks start.

As a means of meeting most of its prospectus objectives as well as the challenge of developing evidence-based research on e-health, PAN has recently been developing its flagship project, PANACeA (PAN Asian Collaborative Evidence-Based eHealth Adoption and Applications). This program will support collaborative research that promotes the evidence-based adoption and application of technologically and socioeconomically appropriate e-health solutions in Asia. It includes 8 projects, involving 8 countries (Bangladesh, India, Indonesia, Mongolia, Pakistan, Philippines, Sri Lanka and Thailand), and is coordinated by the Aga Kahn University in Pakistan with support from the University of Calgary, PrimaCare Malaysia, the Molave Foundation and Angeles University Foundation in the Philippines.

The health sector in Latin America and the Caribbean (LAC) faces a number of key challenges, such as equitable access to health care services, the reduction of costs, and the necessary increase of disease prevention measures among low-income and vulnerable populations, among others. As with Acacia and PAN, the LAC prospectus affirms that digital technologies and ICT-based solutions provide a powerful tool to change the ways in which health services are managed and delivered to the population at large, and to low-income and marginalized communities in particular. ICTs and the Internet, for example, can bring to these communities (at low cost) contacts with larger health centres located in urban areas, opening access to health prevention measures, consultations, updated valuable medical information, coordination in the treatment of patients, adequate and timely distribution of medicines, collection and effective distribution of valuable data on profiles and patterns of threatening epidemics, contagious diseases and other ailments, among others. Attention will also be paid to the relationship between environmental degradation and its impact on the health of the LAC population (see Description of the ICT4D Americas Program Initiative).

**Textbox 1:**
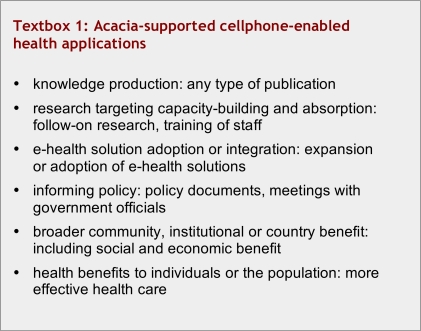
Acacia-supported cellphone-enabled health applications

**Textbox 2:**
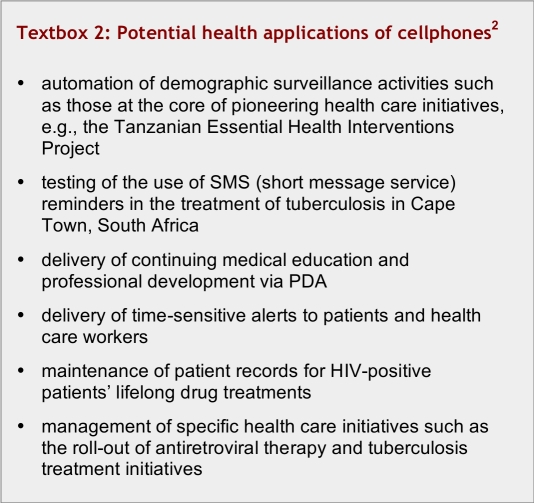
Potential health applications of cellphones

## The future: cautious enthusiasm

We believe that telehealth and e-health solutions can have real, short-term benefits at many levels, including a direct benefit to patients. Reductions in medical error, the realization of costs savings, real-time monitoring of public health incidents and the provision of validated data and information for health systems decision- and policy-making are just some of these benefits.

However, there is an ongoing need to support research that demonstrates these benefits within the framework of a cost–benefit analysis in order to justify the often significant up-front costs associated with the implementation of comprehensive, system-wide telemedicine solutions. This, of course, is particularly significant in the context of developing countries with limited financial resources and telecommunications infrastructure. Although these constraints are limiting in many ways, there are significant opportunities to develop innovative approaches to telemedicine that often do not have to contend with legacy systems and bloated bureaucracies in these environments.

Telemedicine and e-health solutions that are shown to be appropriate, affordable and effective in one region can be adopted in other regions provided they are localized and contextualized. Because significant threats to human health — such as infectious pandemics and geophysical disasters — do not respect political boundaries, these global initiatives carry a sense of urgency.

It should be noted that the failure rate for ICT projects as an industry average is around 50% (link). The fundamental issue that seems to pervade the case histories of failed health ICT projects — a lack of focus on the patient — must be addressed. By putting the patient at the centre and continually verifying that the link between the targeted intervention and the well-being of patients is clear, the likelihood of success will be substantially improved.

We believe that what is now required is the development of a rigorous research methodology that is relevant and applicable to the context of developing nations. Such a methodology must be based on an applied research modality in which the fundamentals of the work address real and significant issues of human health as they influence the development process.

The needs of people living in developing countries are profound. Their pursuit of equity and full participation in global society faces enormous hurdles but, ultimately, is firmly dependent on a healthy society with full access to effective health care. We are committed to finding a way that ICT can achieve this.
